# P057: Epidemiological aspects of MRSA circulation in the industrial region of Russia

**DOI:** 10.1186/2047-2994-2-S1-P57

**Published:** 2013-06-20

**Authors:** EB Brusina, LS Glazovskaya, TV Efimova

**Affiliations:** 1 Department of Epidemiology, Kemerovo State Medical Academy, Kemerovo, Russian Federation

## Introduction

Methicillin-resistant *Staphylococcus aureus* (MRSA) represent one of the major problems related to healthcare-associated infections (HAIs). Investigation of the regional features of MRSA circulation may lead to the improvement of the system of MRSA infection control.

## Objectives

To study features of MRSA circulation in the industrial region of Russia.

## Methods

MRSA identification and determination of susceptibility to antibiotics were performed using VITEK 2 identification cards (colorimetric reading). MRSA DNA was defined using real-time polymerase chain reaction (PCR) with fluorescent hybridization probes. MecA structural gene was determined by sequencing, identification of sea, seb, sec, tst, and pvl genes was performed using PCR. Identification of MRSA clonal profile was investigated by restriction-modification (RM)-test and by spa sequence typing.

## Results

The share of MRSA among all strains of *Staphylococcus aureus* in 2012 was 16.63%, that was almost 2-fold lower compared to 2007 (32.09%), and from 2007 till 2012 a steady decrease of this value was noted. The prevalence of MRSA among healthy population was 13,25 per 1,000. MRSA share among patients with bloodstream infections was the highest, reaching 21.85% (95%CI=15.55-29.3). The lowest MRSA share was registered among patients with genital infections (13.17%, 95%CI=11.1-15.49). In the burn units, the spread of MRSA reached epidemic level (80%). We found that 49.25% of all strains were sea-positive, 85.07% were sec-positive, and 13.43% were tst-positive. No seb- or pvl-positive strains were identified. We also revealed using RM-test that our strains were mainly classified to CC8/239 clonal complex.

## Conclusion

The measures of MRSA infection control should take into account regional features.

## Disclosure of interest

None declared.

